# *EHP* Update

**DOI:** 10.1289/ehp.12402

**Published:** 2009-01

**Authors:** Hugh A. Tilson

**Affiliations:** EHP, E-mail: tilsonha@niehs.nih.gov

January marks the completion of my first year as editor-in-chief of *Environmental Health Perspectives* (*EHP*). Anniversaries and new years always tend to promote reflection on the year just past and provide an opportunity to look toward the future. Readers of *EHP* are no doubt aware of the instability that the journal endured in recent years related to the proposal made in 2005 to privatize this federally funded publication. Fortunately, the negative response of the environmental health science community led to a rejection of that proposal. Future support for the journal was articulated in a congressional hearing in 2007, at which time Samuel H. Wilson, acting director of the National Institute of Environmental Health Sciences (NIEHS), pledged renewed and ongoing support of the institute for the journal and its programs.

As a tool for communication, *EHP* obviously relies on words and images. However, it has been the restoring, renewing, and expanding of the journal’s resources that have demonstrated the institute’s long-term commitment to *EHP* ’s mission to provide the highest-quality and most timely environmental health research and information to people around the world. Over the last several months, many people including the *EHP* staff, interim editors, consulting editors, and NIEHS senior management have worked hard to ensure the integrity of the journal and the quality of its news and science content. The following describes some major milestones met by the journal during the last year.

Year’s end is neither an end nor a beginning but a going on, with all the wisdom that experience can instill in us.

—Hal Borland (1900–1978)

In July 2008, we were able to put into place a renewed 5-year contract to provide production, design, and web-site support for the journal. Given this renewed long-term commitment to the journal, we expect to embark on new and exciting paths for presenting and distributing our content that will take advantage of innovative technologies and the latest trends in scientific publishing.

On the editorial side of the equation, two extremely important activities have already begun to bear fruit for the journal’s continued growth and success. First, *EHP*’s Associate Editor and Editorial Review Boards were reconstituted to maximize our access to and engagement with research leaders across the broad spectrum of scientific fields that contribute to our understanding of environmental health. Second, in July the journal implemented Manuscript Central, an online manuscript management system. The use of this software has already allowed for more diverse and direct involvement by our associate editors in the journal’s peer-review process and resulted in an approximate 20% decrease in time from submission to publication, a boon for both authors and the research community.

In addition to these editorial improvements, *EHP* is pleased to welcome a new science editor to our permanent staff. Jane Schroeder, an epidemiologist who came to *EHP* from the University of North Carolina at Chapel Hill, is poised to reinvigorate our search for the best papers on the most important findings in environmental health research.

Throughout the past year, much attention and effort has been given to restoring several key outreach areas de-emphasized previously due to budget and resource constraints. Some of these were specifically identified during the congressional hearing and include our international and science education programs. With regard to the former, *EHP* has renewed the relationship with Shanghai CDC to translate and distribute selected *EHP* content to a broad audience of environmental scientists and managers in China. In addition, we will continue to work in partnership with *Ciencia & Trabajo*, *Ciência & Saúde Coletiva,* and *Mali Médical*; recently we created a new content-sharing partnership with the Instituto Nacional de Salud Publica in Mexico. We have also redesigned our International Program webpage and resumed translations of selected content into Spanish, Chinese, and French. *EHP* is now in a position to seek new partnerships with other journals and to contribute to improving the infrastructure of other journals by providing access to publication expertise and experience concerning the peer-review process.

We are also excited to announce that science education lesson development and distribution has resumed and has been greeted with great enthusiasm by high school teachers and others in the education community. In a new international effort, we will provide these lessons to Spanish-speaking students in Mexico City. For the future, it is our goal to expand our Science Education Program to reach more educational levels, increase awareness and access to these tools by foreign teachers and students, and engage students using more contemporary learning tools and approaches.

Finally, *EHP* is in the process of redesigning its website. This process has led us to think about ways to improve navigation of the website and upgrade our search engine. Such improvements will help those who use our journal for research and those seeking information to develop and support environmental health policies and practices. We are also thinking about ways to take advantage of technological trends to make information available in formats that are the most engaging and familiar to a younger generation of environmental health scientists and students.

All of the activities described above have taken place with the express support and encouragement of senior management at NIEHS. Such “in-house” support for the journal is gratefully acknowledged and appreciated by the journal. Moreover, as I have met and talked with people throughout the environmental research, public health, and scientific publishing communities over the last year, it has become increasingly clear that the need for *EHP*—and publications like it—has never been greater. More than ever, as we face environmental health problems, including old foes such as toxic exposures and even more daunting new ones such as global climate change, the world’s citizens require open, accessible, and most importantly, credible scientific information from their governments and other public institutions. *EHP* has long been at the forefront of this effort, and I am proud to have the opportunity and responsibility of shepherding our efforts into the future. *EHP* and its staff look forward with great enthusiasm and hopefulness to the next year, and we thank everyone for their continued support.

## Figures and Tables

**Figure:**
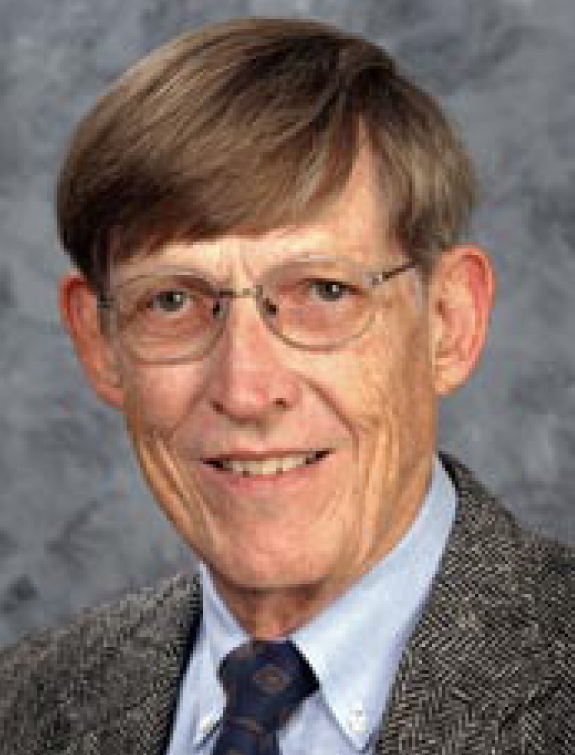
Hugh A. Tilson

